# A two-layer integration framework for protein complex detection

**DOI:** 10.1186/s12859-016-0939-3

**Published:** 2016-02-24

**Authors:** Le Ou-Yang, Min Wu, Xiao-Fei Zhang, Dao-Qing Dai, Xiao-Li Li, Hong Yan

**Affiliations:** College of Information Engineering, Shenzhen University, Shenzhen, 518060 China; Intelligent Data Center and Department of Mathematics, Sun Yat-Sen University, Guangzhou, 510275 China; Institute for Infocomm Research (I2R), A*STAR, 1 Fusionopolis Way, Singapore, Singapore; School of Mathematics and Statistics & Hubei Key Laboratory of Mathematical Sciences, Central China Normal University, Wuhan, 430079 China; Department of Electronic Engineering, City University of Hong Kong, Hong Kong, China

**Keywords:** Protein complex, Protein interaction data, Co-complex matrix, Consensus matrix, Matrix fusion, Matrix decomposition

## Abstract

**Background:**

Protein complexes carry out nearly all signaling and functional processes within cells. The study of protein complexes is an effective strategy to analyze cellular functions and biological processes. With the increasing availability of proteomics data, various computational methods have recently been developed to predict protein complexes. However, different computational methods are based on their own assumptions and designed to work on different data sources, and various biological screening methods have their unique experiment conditions, and are often different in scale and noise level. Therefore, a single computational method on a specific data source is generally not able to generate comprehensive and reliable prediction results.

**Results:**

In this paper, we develop a novel Two-layer INtegrative Complex Detection (TINCD) model to detect protein complexes, leveraging the information from both clustering results and raw data sources. In particular, we first integrate various clustering results to construct consensus matrices for proteins to measure their overall co-complex propensity. Second, we combine these consensus matrices with the co-complex score matrix derived from Tandem Affinity Purification/Mass Spectrometry (TAP) data and obtain an integrated co-complex similarity network via an unsupervised metric fusion method. Finally, a novel graph regularized doubly stochastic matrix decomposition model is proposed to detect overlapping protein complexes from the integrated similarity network.

**Conclusions:**

Extensive experimental results demonstrate that TINCD performs much better than 21 state-of-the-art complex detection techniques, including ensemble clustering and data integration techniques.

**Electronic supplementary material:**

The online version of this article (doi:10.1186/s12859-016-0939-3) contains supplementary material, which is available to authorized users.

## Background

Understanding the structural and functional architecture of the cell has been a fundamental task for systems biology [1]. As vital macromolecules, proteins do not act individually, but work by interacting with other partners [2]. Almost all of the functional processes within a cell are carried out by protein complexes which are formed by interacting proteins [3]. Therefore, detecting protein complexes from protein-protein interaction (PPI) data is crucial for elucidating the modular structure within cells [4, 5]. In recent years, high-throughput screening (HTS) techniques have been designed to detect protein-protein interactions, e.g., yeast two-hybrid (Y2H) [6] and Tandem Affinity Purification/Mass Spectrometry (TAP) [7]. Such HTS techniques have already generated a large amount of PPI data, which facilitate the development of computational methods for protein complex detection [8–21].

Generally, computational methods for protein complex detection utilize two types of data, namely, the binary protein interaction data detected by HTS techniques such as Y2H method, and the data for co-complex interactions among proteins [22, 23] from TAP experiments. Here, we denote the above two types of data as PPI data and TAP data respectively. PPI data is usually modeled as a graph (i.e., PPI network) where nodes represent proteins and edges represent protein interactions. A number of graph clustering algorithms have been proposed for detecting protein complexes from PPI networks, such as MCODE [9], CFinder [24], MCL [8], RNSC [25], COACH [26] and ClusterONE [15]. On the other hand, raw data from TAP experiments is a list of bait proteins along with their corresponding prey proteins that they pulled out (purification records), which could be modeled as a bipartite graph (in which the two node sets are composed of bait proteins and prey proteins, and the edges between the two node sets represent bait-prey connections). Several algorithms have been proposed to identify protein complexes from TAP data as well [27–31]. A common strategy is to first define affinity scores between proteins based on the purification records [5, 32, 33] and then convert the TAP data to a PPI network by using a threshold method to keep those reliable interactions for further analysis. Since convert the original TAP data into a binary PPI network not only introduces errors but also loses useful information in the raw data [23], another alternative strategy is to detect complexes from the TAP data directly, such as CACHET [31].

As diverse sources of protein interaction data are available, data integration becomes a common methodology to reduce the noise in PPI and TAP data (address false positive issue) [34] and to improve the coverage (address false negative issue) for protein complex detection. For example, DECAFF [35] exploited the Gene Ontology (GO) annotations to assess the reliability of PPI data and then detected protein complexes from the refined PPI data; MATISSE [36] and CMBI [37] integrated gene expression data with PPI data to increase the confidence of interactions for protein complex detection. InteHC [17] integrated four data sources (i.e., PPI data, gene expression profiles, GO terms and TAP data) and significantly improved the complex detection. In particular, InteHC calculated a score matrix for each of the four data sources and took their weighted sum, which relies on additional supervision information to learn a weight for each data source, as an integrated matrix. However, due to noise in different data sources, the direct fusion of several original datasets may exacerbate the problems of noise. Moreover, how to correctly estimate the co-complex relationships between proteins based on their functional annotations and gene expression profiles is still an open problem.

Nevertheless, with various methods proposed for protein complex detection, we are thus able to generate a series of clustering results for each type of data. Clearly, given one type of data, each method has its own advantages and limitations in capturing co-complex relationships between proteins [38]. Ensemble clustering, which exploits the complementary nature of individual methods by leveraging their clustering outputs, is thus promising to improve the detection for protein complexes [18, 39, 40]. Particularly, ensemble clustering methods usually first reconstruct a consensus matrix (or consensus network) which shows the co-complex propensity among proteins from a series of clustering results and then apply a specific algorithm [18, 41] to detect protein complexes from the consensus matrix. However, the consensus network, based solely on the result-level integration (integrate the clustering results of different methods on a single type of data), may miss the underlying complex information which exist in other types of data. It is thus necessary to combine the consensus matrices derived from multiple types of data to generate a more comprehensive and reliable co-complex similarity matrix, which may facilitate the detection of protein complexes.

Different from Y2H experiments that are prone to identify direct physical interactions, TAP experiments already provide useful information about protein complexes and TAP data describe the co-complex propensity among proteins. However, as TAP data cannot be converted into co-complex interactions in a straightforward manner, several scoring methods have been proposed to estimate the affinity scores between proteins based on the purification records (e.g., bait-prey and prey-prey relationships) provided by TAP data, such as C2S scores [30]. As such, we are able to integrate heterogeneous matrices, i.e., the consensus matrices derived from different types of data and the co-complex score matrices derived from TAP data, to better understand the co-complex relationships among proteins. However, as these heterogeneous matrices may have different scales, noise rates and importance levels, focusing only on common patterns can miss valuable complementary information. It would be challenging to merge them into a final co-complex matrix automatically in an unsupervised manner. In addition, once we obtain the integrated matrix, it is still difficult for us to design an efficient algorithm to detect overlapping complexes from this integrated matrix.

**Fig. 1:**
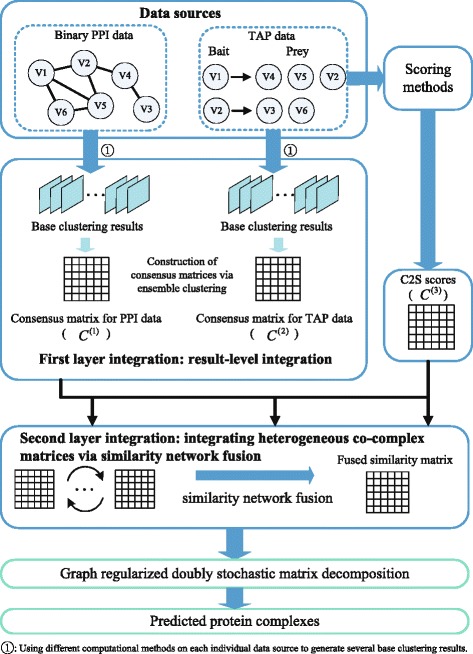
Schematic overview of our proposed TINCD model. TINCD first constructs two consensus matrices (*C* ^(1)^ and *C* ^(2)^) based on the complex knowledge discovered by various graph clustering algorithms from PPI and TAP data. Second, a similarity network fusion (SNF) strategy is employed by TINCD to combine these two consensus matrices with the score matrix obtained from TAP data (*C* ^(3)^) to obtain a final co-complex score matrix. Finally, a novel graph regularized doubly stochastic matrix decomposition model is proposed to detect overlapping protein complexes from the final score matrix

To address the above challenges, we propose a novel Two-layer INtegrative Complex Detection (TINCD) model to predict protein complexes as shown in Fig. 1, which leverages the information from both clustering results and raw data sources. In the first layer integration, we utilize an ensemble method to construct consensus matrices for different types of data to measure the co-complex propensities between proteins based on various clustering results. In the second layer integration, we combine the consensus matrices derived from different types of data with the score matrix derived from TAP data and obtain an integrated similarity network via a similarity network fusion (SNF) method. SNF is an iterative process to fuse heterogeneous networks or matrices by capturing both shared and complementary information among them [42]. Finally, a novel graph regularized doubly stochastic matrix decomposition model is proposed to detect overlapping protein complexes from the integrated similarity network. We have conducted comprehensive experiments to evaluate the performance of our proposed TINCD algorithm. A comprehensive comparison with 21 existing methods shows that our two-layer integration strategy generates protein complexes with better coverage and accuracy. All the experimental results and code can be downloaded from https://github.com/Oyl-CityU/TINCD.

## Methods

In this section, we describe our TINCD model as shown in Fig. 1 in details. We first demonstrate the two-layer integration and then present the graph regularized doubly stochastic matrix decomposition algorithm for protein complex detection.

### First layer integration: result-level integration via ensemble clustering

As diverse types of data are available and various computational methods have been designed to detect protein complexes from them, we are thus able to generate a series of base clustering results (i.e., employing different methods on a particular type of data will generate multiple clustering results). A straightforward way to measure the co-complex affinities among proteins is to build the consensus matrices by integrating the above abundant clustering results.

Suppose all the data sources used in this study cover *N* proteins and we have obtained *n*_*p*_ clustering results which are generated by applying *n*_*p*_ different methods on a specific type of data. Here, a clustering result refers to a set of clusters generated by a certain method. A consensus matrix *C*^(*m*)^ is a *N*×*N* matrix. In *C*^(*m*)^, the entry $C_{\textit {ij}}^{(m)}$ is the number of clustering results where the proteins *i* and *j* are assigned to the same cluster, divided by the number of clustering results *n*_*p*_. As such, each entry $C_{\textit {ij}}^{(m)}$ indicates the probabilities that protein *i* and *j* are involving in the same complexes. If protein *i* is not assigned to any clusters or is not included in the *m*-th data source, the *i*-th row and *i*-th column of *C*^(*m*)^ are set to zero. Note that the coverage and quality of different data sources would be different. We thus build a corresponding consensus matrix independently for each type of data. In this study, we focus on two data sources, namely, PPI data and TAP data. Therefore, the consensus matrices corresponding to these two types of data are denoted by *C*^(1)^ and *C*^(2)^ respectively (please refer to Fig. 1).

### Second layer integration: integrating heterogeneous co-complex matrices via similarity network fusion

Unlike PPI data, the TAP data is designed to capture the co-complex relationships between proteins. In addition to the above consensus matrices, we also calculate an affinity score matrix to capture the co-complex relationships directly from the TAP data (in the raw data level). For TAP data, several scoring methods have been proposed to measure the affinity between proteins based on the purification records [5, 30, 32, 33]. For example, C2S method [30] has been recently developed for measuring the co-complex relationships among proteins. In this paper, we further process the C2S scores by discarding the negative scores (according to the definition of C2S scores, protein pairs with negative scores are not likely to be co-complex) and taking exponential transformation for the positive scores (normalized to [0,1] from original values), and generate a matrix *C*^(3)^. Assume that *C*2*S*_*ij*_ is the original score between the proteins *i* and *j*, $C_{\textit {ij}}^{(3)}$ is our refined score as follows.

(1) $$ C_{ij}^{(3)} = \left\{ \begin{array}{cc} 1- \exp(-C2S_{ij}), & \text{if}\,\, C2S_{ij} > 0, \\ 0, & \text{if}\,\, C2S_{ij} \leq 0. \end{array} \right.  $$

Let *C*^(*m*)^ (*m*=1,…,*M*) denote all the consensus matrices from the ensemble clustering and the score matrix derived from the TAP data (in this study, *M*=3). All of these *M* matrices describe the co-complex similarities among proteins, but in different scales and with different noise rates. We next introduce the similarity network fusion (SNF) method [42] to integrate these *M* heterogeneous matrices.

After defining similarity matrices *C*^(*m*)^, the normalized weight matrices *A*^(*m*)^ are defined in Eq. (2). The normalization here is free of the scale of self-similarity in the diagonal entries and avoids numerical instabilities and it satisfies $\sum _{j} A_{\textit {ij}}^{(m)} = 1$.

(2) $$ A_{ij}^{(m)} = \left\{ \begin{array}{cc} \frac{C_{ij}^{(m)}}{2\sum_{v\neq i} C_{iv}^{(m)}} & \text{if}\,\, j \neq i, \\ \frac{1}{2}, & \text{if}\,\,j = i \end{array} \right.  $$

Local neighborhoods are further exploited to measure the local affinities among proteins. Let $V_{i}^{(m)}$ denote the *L* (the value of *L* is set to be 20 by default in [42]) nearest neighbors of protein *i* in the matrix *C*^(*m*)^ (*m*=1,…,*M*). To measure the local affinity, the local kernel matrix *B*^(*m*)^ is defined in Eq. (3). By this operation, the *L* most similar proteins for each protein are kept and those neighbors with low similarities are filtered out. Therefore, *B*^(*m*)^ captures the local structure of similarity network corresponding to *C*^(*m*)^. Overall, *A*^(*m*)^ carries the full information about the similarity of each protein to all the others, while *B*^(*m*)^ only encodes the similarity to nearby proteins.

(3) $$ B_{ij}^{(m)} = \left\{ \begin{array}{cc} \frac{C_{ij}^{(m)}}{\sum_{v\in V_{i}^{(m)}} C_{iv}^{(m)}} & \text{if}\,\, j \in V_{i}^{(m)},\\ 0, & \text{otherwise} \end{array} \right.  $$

Let $W_{t=0}^{(m)} = A^{(m)} (m=1, \ldots, M$) represent the initial status matrices at *t*=0. SNF is an iterative process to update the status matrix *W*^(*m*)^ in Eq. (4) as follows:

(4) $$ W_{t+1}^{(m)} = B^{(m)} \times \left(\frac{1}{M-1} \sum\limits_{v \neq m} W_{t}^{(v)}\right) \times \left(B^{(m)}\right)^{T}.  $$

Another way to think of the updating rule (4) is:

(5) $$ \begin{aligned} {} W_{t+1}^{(m)} (i,j)& = \sum\limits_{h\in V_{i}^{(m)}}\sum\limits_{l \in V_{j}^{(m)}} B_{i,h}^{(m)}\\ &\quad\times \left(\frac{1}{M-1} \sum\limits_{v \neq m} W_{t}^{(v)}\right)_{h,l}\times B_{j,l}^{(m)}. \end{aligned}  $$

Note $V_{i}^{(m)}$ represents the neighborhood of protein *i* in matrix *C*^(*m*)^ (*m*=1,…,*M*). If proteins *i* and *j* have common neighbors in *C*^(*m*)^ or their neighbors in *C*^(*m*)^ have high similarity scores in other similarity matrices, their co-complex similarity will be augmented through these cross diffusion processes and vice versa. Therefore, even if protein *i* and *j* are not very similar in one data type, their similarity can be expressed in other data types, and this similarity information can be propagated through the fusion process. We perform normalization on $W_{t+1}^{(m)}$ as in Eq. (2) after each iteration [42]. After *t* steps (*W*^(*m*)^ are converged), we can automatically obtain the integrated similarity matrix *W* that fuses all these heterogeneous matrices in Eq. (6) where *M* is 3. Since *A*^(*m*)^ is normalized and *B*^(*m*)^ retains the local similarities (*m*=1,…,*M*), the cross diffusion processes in Eq. (4) are free of the scale and robust to the noise. Hence, the final similarity scores encoded in *W* would be more comprehensive and reliable.

(6) $$ W = \frac{1}{M} \sum\limits_{m=1}^{M} W_{t}^{(m)}.  $$

### Detecting protein complexes via graph regularized doubly stochastic matrix decomposition model

In the above sections, we obtain the integrated similarity matrix *W* via a two-layer integration framework. Next, we present the graph regularized doubly stochastic matrix decomposition model to detect protein complexes from *W*.

#### Model formulation

Our objective is to infer *P*(*k*|*i*) from *W*, which is the probability of assigning protein *i* to the predicted *k*-th complex. If we cluster all proteins into *K* complexes, the complex assigning probabilities represent the single-step random walk probabilities from proteins to complexes. Without preference to any particular proteins, we impose uniform prior *P*(*i*)=1/*N* over the proteins. In this way, the reversed random walk probabilities can be calculated by the Bayes formula:

$$P(i|k) = \frac{P(k|i)P(i)}{\sum_{z=1}^{N} P(k|z)P(z)} = \frac{P(k|i)}{\sum_{z=1}^{N} P(k|z)}. $$

Taking into account the probability of two-step random walks from protein *i* to protein *j* via all complexes:

$$P(i|j) = \sum\limits_{k=1}^{K} P(i|k)P(k|j) = \sum\limits_{k=1}^{K} \frac{P(k|i)P(k|j)}{\sum_{z=1}^{N} P(k|z)}. $$

This probability defines the similarity between two proteins, $\hat {W}_{\textit {ij}}=P(i|j)$, according to their memberships with respect to all complexes. Note that *W* represents the observed similarity between proteins that arise from their co-complex relationships, the learning target is to find a good approximation between *W* and $\hat {W}$. In this study, we use generalized Kullback-Leibler (KL) divergence, which is suitable for the approximation $W\approx \hat {W}$, to measure the difference between $\hat {W}$ and *W* [43]. The objective function is defined as the following optimization problem:

(7) $$ \left\{ \begin{array}{ll} \min\limits_{\theta \geq 0} & D_{KL}(W||\hat{W}) = \sum_{ij}\left(W_{ij} \log \frac{W_{ij}}{\hat{W_{ij}}} - W_{ij} + \hat{W}_{ij}\right). \\ s.t. & \,\,\sum_{k=1}^{K} \theta_{ik}=1, i = 1,\ldots,N. \end{array} \right.  $$

where *θ*_*ik*_=*P*(*k*|*i*) and $\hat {W}_{\textit {ij}}=\sum _{k=1}^{K} \frac {\theta _{\textit {ik}}\theta _{\textit {jk}}}{\sum _{z=1}^{N} \theta _{\textit {zk}}}$.

Moreover, as each element *W*_*ij*_ of the similarity matrix *W* reflect the observed co-complex similarity between protein *i* and *j*, protein pairs with high value of *W*_*ij*_ are supposed to have similar propensities to be involved in the same complexes. As a popular manifold learning method, graph Laplacian is widely used in semi-supervised learning to enforce smooth regularization for nodes with high similarities [44]. Given the similarity matrix *W*, the Laplacian regularizer for the value of *θ* is defined as follows:

(8) $$ \begin{array}{ll} R &= \frac{1}{2} \sum\limits_{i=1}^{N} \sum\limits_{j=1}^{N} W_{ij} \sum\limits_{k=1}^{K} \left(\theta_{ik} - \theta_{jk}\right)^{2} \\ &= Tr\left(\theta^{T} D \theta\right) - Tr\left(\theta^{T} W \theta\right). \end{array}  $$

where *Tr*(·) denotes the trace of a matrix and *D* is a diagonal matrix defined by $D_{\textit {ii}} = \sum _{j=1}^{N} W_{\textit {ij}}$. By minimizing *R*, we wish the co-complex relationships inherent in *W* could transfer to the estimator of *θ*.

#### Graph regularized doubly stochastic matrix decomposition model

Taking into account the above two factors in Eqs. (7) and (8), and dropping those constants, we present a novel Graph regularized Doubly Stochastic Matrix Decomposition model with the following objective function:

(9) $$ \left\{ \begin{array}{ll} \min\limits_{\theta \geq 0} & \mathcal{J}(\theta) = \sum_{ij}\left(- W_{ij} \log \hat{W_{ij}} + \hat{W}_{ij}\right) \\ & + \lambda \left(Tr(\theta^{T} D \theta) - Tr(\theta^{T} W \theta)\right)\\ s.t. & \,\,\sum_{k=1}^{K} \theta_{ik}=1, i = 1,\ldots,N. \end{array} \right.  $$

where *λ*≥0 is the tradeoff parameter that controls the balance between the two factors.

Since the above objective function (9) is non-convex, we employ a relaxed Majorization-Minimization algorithm to find a good local minima [43]. The update rule for *θ* is shown in Algorithm ??. Please refer to Additional file 1 for more details. Since the optimal solution $\hat {\theta }_{\textit {ik}}$ is a continuous value which describes the probability of assigning protein *i* to the predicted *k*-th complex, we need to discretize $\hat {\theta }$ into a final protein-complex assignment matrix *θ*^⋆^. In this study, to get overlapping protein complexes, for each protein *i*, we first sort $\hat {\theta }_{\textit {ik}}$, *k*=1,…,*K* in descending order, then we retain the top *K*_*i*_ complexes if the gap between the *K*_*i*_-th and (*K*_*i*_+1)-th element is the largest. ${\theta }^{\star }_{\textit {ik}}=1$ if *k* belongs to the top *K*_*i*_ complexes, and ${\theta }^{\star }_{\textit {ik}}=0$ otherwise.



Here, ${\theta }^{\star }_{\textit {ik}} = 1$ represents protein *i* is assigned to the predicted *k*-th complex while ${\theta }^{\star }_{\textit {ik}} = 0$ indicates protein *i* does not belong to the predicted *k*-th complex. In this study, we only consider predicted complexes with at least three proteins [15].

## Results

In this section, we first introduce the experiment settings, i.e., experiment data and evaluation metrics. Then, we demonstrate an extensive comparison study between our proposed TINCD method and various existing approaches for protein complex detection.

### Experiment data and evaluation metrics

In this study, two types of data (PPI data and TAP data) for yeast have been employed for evaluating the performance of various complex detection methods. The binary PPI data is downloaded from the DIP database [45], which involves with 17,201 interactions among 4,930 proteins. In addition, we consolidate the data from both [5] and [46] as our TAP data, which consist of 6,498 purifications involving 2,996 bait proteins and 5,405 prey proteins. Overall, the PPI data and TAP data cover 5,929 proteins.

We employ 3 benchmark complex sets as gold-standard to evaluate the complexes predicted by various methods, namely CYC2008 [47], MIPS [48] and SGD [49]. In particular, CYC2008 consists of 408 complexes, MIPS with 203 and SGD with 323, respectively. For all the reference sets, to avoid selection bias, we filter out the proteins that are not involved in the input PPI and TAP data. Moreover, we only consider complexes with at least 3 proteins as suggested by Nepusz et al. [15].

We utilize the *sensitivity* (Sn), *positive predictive value* (PPV), *Accuracy* (Acc) [30] and *FRAC* [15] to evaluate the predicted protein complexes. Given a benchmark complex *x*_*i*_ and a predicted complex *y*_*j*_, the Sn and PPV are defined in Eq. (10), and *Accuracy* is the geometric mean of Sn and PPV. Using *Accuracy* is better than Sn and PPV individually, as it can provide a balanced view of the prediction performance.

(10) $$\begin{array}{@{}rcl@{}} && Sn = \frac{\sum_{i} \max_{j} T_{i, j}}{\sum_{i}|x_{i}|}, PPV = \frac{\sum_{j} \max_{i} T_{i, j}}{\sum_{j} |\cup_{i}(x_{i} \cap y_{j})|}, \\ && Accuracy = \sqrt{Sn \times PPV}  \end{array} $$

where *T*_*i*,*j*_ is the number of proteins shared by *x*_*i*_ and *y*_*j*_, i.e., $|x_{i} \cap y_{j}|$. Fraction of matched complexes (*FRAC*) [15] is an indicator for prediction coverage, which measures the percentage of benchmark complexes that are matched by the predicted complexes. Given *x*_*i*_ and *y*_*j*_, we consider them to be matching if $\frac {|x_{i}\cap y_{j}|^{2}}{|x_{i}||y_{j}|} \geq \omega $ (Similar to majority of the detection methods, we set *ω* as 0.2 in our experiments). FRAC is defined in Eq. (11), where *X* is the set of benchmark complexes and *Q* is the set of predicted complexes.

(11) $$ FRAC = \frac{\left|\left\{x_{i}|x_{i}\in X \wedge \exists y_{j}\in Q, y_{j}\,\,matches\,\,x_{i}\right\}\right|}{|X|}.  $$

### Parameter settings

There are two parameters *K* and *λ* in our model, where *K* is the number of possible complexes and *λ* controls the effects of the Laplacian regularizer. Since we usually do not have any prior knowledge about the number of complexes in real-world situations, it is hard to decide the value of *K*. Fortunately, we have introduced a graph regularization to force proteins with high co-complex similarity scores to have similar memberships. By controlling the effect of this regularization term, we may be able to filter out the irrelevant dimensions of *θ*. If so, we can fit our model with a large value of *K* as our model is able to determine the number of complexes adaptively. Therefore, to test how these two parameters affect the performance of our model, we have performed the sensitivity studies. Particularly, we consider all combinations of the following values: {1500,2000,2500} for *K* and {2^−5^,2^−4^,…,2^7^} for *λ*, and assess how well the complexes predicted by our model match with reference sets.

**Fig. 2:**
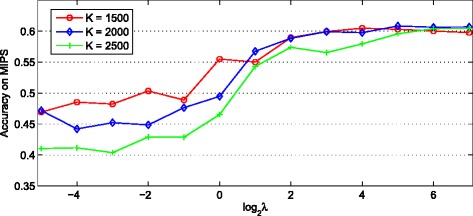
The effect of *λ* and *K*. Performance of TINCD on protein complex detection with different values of *λ* and *K* measured by *Accuracy* with respect to MIPS gold standard. The *x*-axis denotes the value of $\log \lambda $, the *y*-axis denotes the value of *Accuracy*

The performance of TINCD is measured by *Accuracy* with respect to MIPS gold standard. As shown in Fig. 2, for a fixed value of *K*, as the value of *λ* increases, the value of *Accuracy* fluctuates slightly in the beginning and then increases steadily until converge. Overall, TINCD obtains competitive *Accuracy* scores when *λ*∈[2^5^,2^7^]. On the other hand, when the value of *λ* is less than 2, the larger the value of *K*, the worse the effect of TINCD. We can also find that with the increase of the value of *λ*, the influence of *K* is waning. A possible reason would be that we use graph regularization to force proteins with high co-complex similarity scores to have similar memberships. When the value of *λ* is large enough, irrelevant latent indexes always obtain lower associations. Therefore, TINCD is not very sensitive to the value of *K* when *λ* is large enough. In this case, the value of *K* could be relatively large since irrelevant clusters will be automatically filtered out. Based on the above sensitivity analysis as shown in Fig. 2, *K*=2000 and *λ*=2^5^ would be the optimal setting for parameters *K* and *λ* on MIPS data. To avoid overestimating the performance of TINCD, we will also set *K*=2000 and *λ*=2^5^ as the default values on other benchmark sets (e.g., CYC2008).

### Similarity network fusion (SNF) vs. matrix averaging

In the experiments, the consensus matrices are built via integrating various base clustering results from PPI data and TAP data. In particular, 11 state-of-the-art approaches are applied to PPI data to generate complexes, including CFinder [24], CMC [50], COACH [26], ClusterONE [15], DPClus [51], IPCA [52], MCL [8], MCODE [9], RNSC [25], RRW [53] and SPICi [54]. In this study, optimal parameters are set for CFinder, CMC, COACH, DPClus, IPCA, MCL, MCODE, RRW and SPICi to generate their best results while ClusterONE and RNSC have used the default parameters set by the authors. For detailed parameter settings of these algorithms, please refer to Additional file 1. The consensus matrix based on these 11 base clustering solutions is denoted as **P**. We also collect the complexes predicted from TAP data by 5 existing methods, including BT [29], C2S [30], CACHET [31], Hart [27] and Pu [28]. Protein complexes predicted by these 5 methods are downloaded from http://www.ntu.edu.sg/home/zhengjie/data/InteHC/. The consensus matrix based on these 5 solutions for TAP data is denoted as **T**. In addition, **P+T** denotes the combination of two consensus matrices **P** and **T**. SNF is thus applied to integrate the C2S matrix with the consensus matrices (e.g., **P**, **T** and **P+T**). In addition, a natural way to integrate these matrices is to take an average for them, and we denote this method as Matrix Averaging. Next, we will take Matrix Averaging as baseline and compare it with the SNF method.

**Fig. 3:**
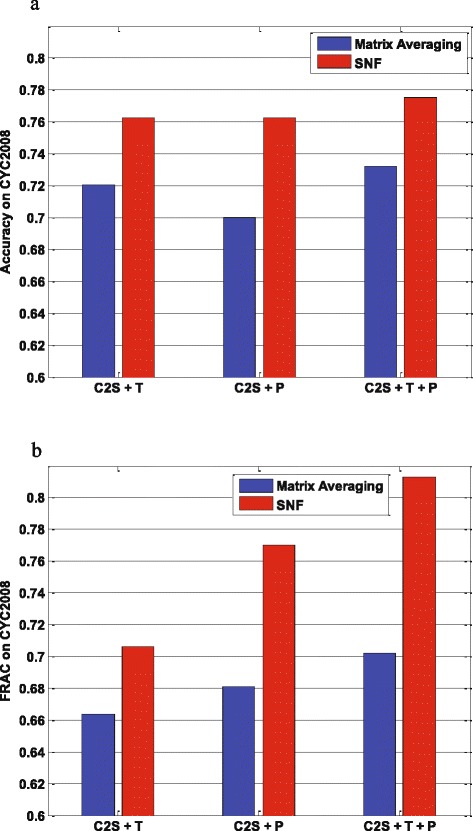
SNF vs. Matrix Averaging. Performance of SNF and Matrix Averaging in terms of **a** *Accuracy* and **b** FRAC with respect to CYC2008

Figure 3 shows the performance of our TINCD with the fused similarity matrix generated by SNF and Matrix Averaging, in terms of Accuracy and FRAC with respect to CYC2008. SNF performs consistently better than Matrix Averaging when we combine C2S matrix with **T**, **P** and **T+P**, respectively. The reason is that simple fusion techniques such as Matrix Averaging are sensitive to the noise in the data, while SNF as a cross diffusion process is robust to the noise. More importantly, SNF can capture both shared and complementary information from the heterogeneous matrices. We obtained similar results evaluated on two other benchmarks MIPS and SGD (Additional file 1: Figures S1 and S2) and please refer to Additional file 1 for more details.

Moreover, we have two observations by comparing the performance of different consensus matrices as shown in Fig. 3.

Firstly, integrated with C2S matrix via SNF, the consensus matrix **P** performs much better than **T**. For example with reference data CYC2008, **C2S+P** and **C2S+T** obtain comparable *Accuracy*, while **C2S+P** has a higher FRAC than **C2S+T** (0.770 for **C2S+P** vs. 0.706 for **C2S+T**). The rationale behind this finding would be that **T** is redundant with C2S to some extent (both from TAP data), while **P** complements C2S well (PPI and TAP) to achieve better performance.

Secondly, by adding **T** to **C2S+P**, **C2S+P+T** achieves better performance than **C2S+P**. Comparing **C2S+P** with respect to CYC2008, the *Accuracy* of **C2S+P+T** is increased by 1.7 % from 0.763 to 0.776 while its FRAC is increased by 5.58 % from 0.770 to 0.813. As shown in Additional file 1, both *Accuracy* and FRAC of **C2S+P+T** are improved on SGD benchmark complexes, i.e., the *Accuracy* improves by 4.1 % from 0.711 to 0.740 and the FRAC increases by 9.4 % from 0.678 to 0.742. Overall, we would think that **C2S+P+T** performs better than **C2S+P** and **C2S+T**, and our TINCD refers to the clustering over **C2S+P+T** thereafter.

### Clustering the integrated matrix

Once we obtained the integrated matrix (i.e., **C2S+P+T**), we are able to apply various clustering methods to generate protein complexes in our framework, e.g., Nonnegative Matrix Factorization (NMF) and Agglomerative Hierarchical Clustering (HC). Since the integrated matrix corresponds to a weighted network, and only few methods can deal with large scale weighted networks. In this section, we will compare our proposed graph regularized doubly stochastic matrix decomposition model with NMF, HC, ClusterONE and SPICi. All of these four algorithms are able to detect complexes from weighted PPI networks directly and output the results in a reasonable time. In particular, NMF is a popular clustering algorithm which can be related to a generalized form of many clustering methods (i.e., Kernel K-means clustering and spectral clustering.) [55]. In this study, NMF is solved by DTU:Toolbox [56] via multiplicative update method. For HC, it first considers all singleton proteins as initial clusters, then it iteratively merges two clusters with the highest similarity in each iteration. The iterative algorithm terminates when quality function of the detected clusters has achieved its maximal value. Similar to [17], three quality functions are used to measure the quality of a set of clusters, the corresponding results are thus denoted by HC-Q1, HC-Q2 and HC-Q3 respectively. For more details about these three quality functions, please refer to [17]. For a fair comparison, optimal parameters are set for these four algorithms to generate its best results (For NMF, the number of clusters is chosen from 1000 to 2000 with 100 as increment. For SPICi, we try different values of density threshold, ranges from 0.1 to 1 with 0.1 as increment. ClusterONE has used the default parameters set by the authors.).

**Fig. 4:**
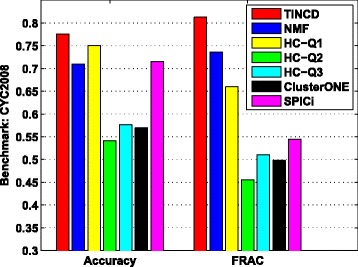
Clustering the integrated matrix. *Accuracy* and FRAC of TINCD, NMF, Hierarchical clustering with 3 different quality functions (i.e., HC-Q1, HC-Q2 and HC-Q3), ClusterONE and SPICi with respect to CYC2008

Figure 4 shows the *Accuracy* and FRAC of TINCD, NMF, HC with various quality functions (i.e., HC-Q1, HC-Q2 and HC-Q3), ClusterONE and SPICi. We observe in Fig. 4 that TINCD performs better than NMF, HC-Q1, HC-Q2, HC-Q3, ClusterONE and SPICi. For example, the *Accuracy* of TINCD with respect to CYC2008 is 0.776, which is 3.5 % higher than the second best *Accuracy* 0.750 achieved by HC-Q1. In addition, the FRAC of TINCD with respect to CYC2008 is 0.813, which is 10.5 % higher than the second best FRAC 0.736 achieved by NMF. The integrated similarity network describes the probabilities of random walks from each protein to other proteins based on their co-complex relationships, which is consistent with the model assumption of our proposed graph regularized doubly stochastic matrix decomposition model. Thus, our TINCD could more accurately discover the complex information from the integrated similarity network (similar results obtained with respect to MIPS and SGD benchmarks are shown in Additional file 1: Figure S3).

### Comparisons with base clustering solutions

As introduced above, we collected 16 base solutions (11 for PPI data and 5 for TAP data) to generate protein complexes. Next, we compare TINCD with these 16 base solutions in terms of their *Accuracy* and FRAC over 3 benchmark complex sets.

Table 1 demonstrates the comparison between TINCD and 16 base solutions with respect to CYC2008. For example, DPClus and C2S achieve the highest FRAC 0.680 and 0.664 among the base solutions for PPI data and TAP data, respectively. TINCD achieves a FRAC 0.813, which is 19.6 % and 22.4 % higher than DPClus and C2S. In addition, COACH achieves the highest *Accuracy* 0.650 among PPI base solutions while C2S is 0.761. Thus, TINCD with Accuracy 0.776 is 2.0 % and 19.4 % higher than C2S and COACH, respectively. Overall, TINCD performs much better than all the base solutions in terms of both FRAC and Accuracy (similar results obtained with respect to MIPS and SGD benchmark are shown in Additional file 1: Table S1).

**Table 1:** Comparison between TINCD and state-of-the-art methods with respect to CYC2008

Methods	No. of complexes	No. of covered proteins	Acc	FRAC
TINCD	1562	5846	0.776	0.813
EC-BNMF	457	2105	0.751	0.677
CMBI	618	1041	0.459	0.349
InteHC	684	3400	0.748	0.634
CFinder	245	2008	0.518	0.319
CMC	562	1651	0.643	0.655
COACH	746	1838	0.650	0.664
ClusterONE	342	1366	0.584	0.438
DPClus	651	2140	0.639	0.680
IPCA	816	1621	0.617	0.575
MCL	600	4101	0.644	0.536
MCODE	108	666	0.485	0.311
RNSC	541	2095	0.619	0.506
RRW	248	1174	0.571	0.511
SPICi	412	2113	0.607	0.502
BT	409	1286	0.728	0.591
C2S	1035	4500	0.761	0.664
CACHET	449	964	0.674	0.553
Hart	390	1307	0.720	0.600
Pu	400	1504	0.732	0.579

An observation in Table 1 is that 5 base solutions for TAP data are much better than those 11 base solutions for PPI data. The consensus matrix **P** generated by these weaker base solutions for PPI data, however, performs much better than **T** as shown in Fig. 3. This observation highlights once again that the consensus matrix **P** from PPI data is a good complement to C2S score matrix for protein complex detection.

### Comparison with ensemble clustering

**Fig. 5:**
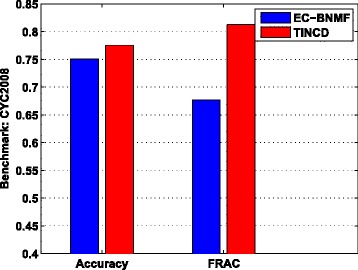
Comparison with ensemble clustering. *Accuracy* and FRAC of TINCD and EC-BNMF with respect to CYC2008

We further compared TINCD with EC-BNMF (Ensemble Clustering via Bayesian Nonnegative Matrix Factorization), which generated ensemble clusters from the above 16 base clustering solutions. For a fair comparison, optimal parameters are set for EC-BNMF to generate its best results. For detailed parameter settings of EC-BNMF, please refer to Additional file 1. Figure 5 shows the *Accuracy* and FRAC of TINCD and EC-BNMF with respect to CYC2008.

In Fig. 5, TINCD achieves higher *Accuracy* than EC-BNMF (0.776 for TINCD vs. 0.751 for EC-BNMF). In addition, TINCD achieves a FRAC 0.813, which is 20.09 % higher than EC-BNMF (0.677). Hence, TINCD outperforms the ensemble clustering method EC-BNMF in terms of both Accuracy and FRAC (similar results obtained with respect to MIPS and SGD benchmarks are shown in Additional file 1: Table S1).

### Comparison with data integration techniques

In addition to ensemble clustering techniques which integrate clustering results, another type of integrative techniques aims to integrate diverse data sources for protein complex detection. For example, CMBI integrates PPI data, gene expression profiles and essential protein information to detect protein complexes, while InteHC integrates PPI data, TAP data, gene expression profiles and gene ontology annotations for protein complex prediction. Next, we compare our TINCD with data integration techniques CMBI and InteHC. Protein complexes predicted by CMBI and InteHC are downloaded from http://www.ntu.edu.sg/home/zhengjie/data/InteHC/.

**Fig. 6:**
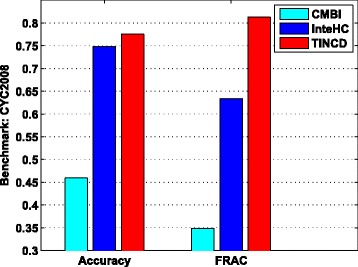
Comparison with data integration techniques. *Accuracy* and FRAC of CMBI, InteHC and TINCD with respect to CYC2008

Figure 6 shows the *Accuracy* and FRAC of CMBI, InteHC and TINCD with respect to CYC2008. Both InteHC and TINCD perform much better than CMBI, and we then focus on the comparison between InteHC and TINCD. Overall, TINCD outperforms InteHC with respect to CYC2008. For example, the *Accuracy* and FRAC of TINCD with respect to CYC2008 are 0.776 and 0.813, which are 3.7 % and 28.2 % higher than that of InteHC, respectively (similar results obtained with respect to MIPS and SGD benchmarks are shown in Additional file 1: Table S1).

InteHC integrates various data sources and utilizes some supervision information to assign them different weights according to their importance. Among various raw data sources, TINCD integrates only the C2S scores with consensus matrices in an unsupervised manner and thus is more preferable. The overall better results achieved by TINCD in the more challenging unsupervised setting demonstrate that TINCD is able to achieve better FRAC (by two layer integration), at the same time to maintain a high *Accuracy*. In the future, it would be promising if we integrate more data sources (e.g., gene ontology annotations) into our TINCD framework.

### A case study: the FBP degradation complex

**Fig. 7:**
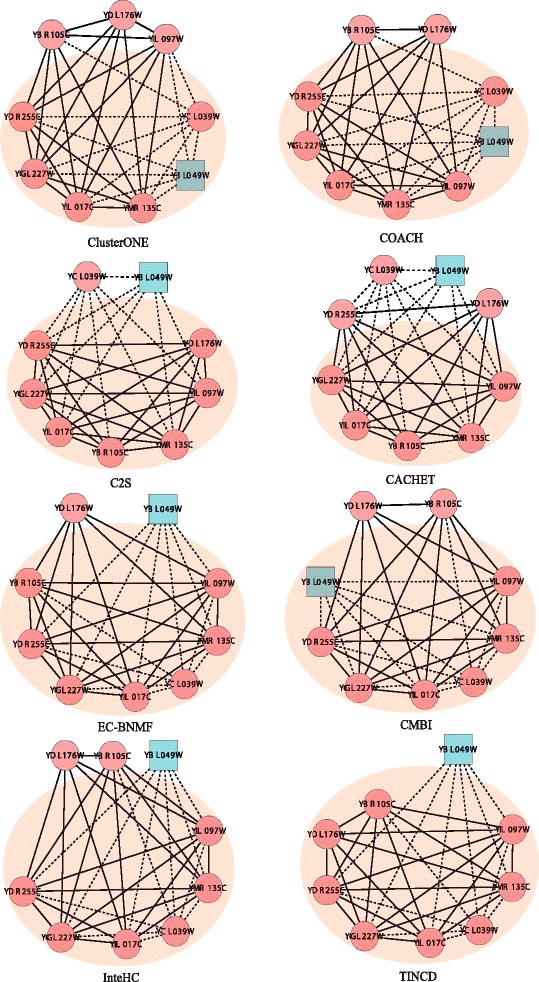
Examples of benchmark protein complexes predicted more accurately by TINCD. The FBP degradation complex as detected by different computational methods. The shadow area shows the complex predicted by each method, red circle nodes represent subunits of the FBP degradation complex in CYC2008, and blue rectangle nodes represent proteins belong to other complexes. In addition, dash lines represent physical interactions and solid lines refer to the pairs with positive C2S scores

Figure 7 shows how the FBP degradation complex is found by the clustering algorithms we have studied. This complex in CYC2008 involves 8 proteins. Proteins that have binary interactions are connected by dash lines, while proteins that do not have binary interactions but have positive C2S scores are connected by solid lines. TINCD is the only algorithm that could correctly cover all the proteins in this complex. All other algorithms make various mistakes as follows. First, ClusterONE and COACH are designed to detect protein complexes from PPI data (binary interactions). They are only able to detect part of the whole complex (i.e., ClusterONE missed 3 proteins while COACH missed 2) and both of them misclassify the protein YBL049W into the FBP degradation complex. Second, C2S and CACHET are designed to detect complexes from TAP data. Similarly, they are only able to detect part of the whole complex, e.g., C2S missed 1 protein while CACHET missed 3. Third, CMBI, EC-BNMF and InteHC are designed to integrate either different clustering results or diverse data sources for protein complex detection. They missed 2, 1 and 2 proteins in the FBP degradation complex respectively. For more examples, see Additional file 1.

## Discussions and conclusions

In this work, we have proposed a novel two-layer integration framework TINCD to identify protein complexes. First, TINCD constructs consensus matrices for proteins and measures their co-complex propensity based on the complex knowledge discovered by various graph clustering results. Second, a similarity network fusion (SNF) strategy is employed by TINCD to combine consensus matrices and score matrix obtained from TAP data to obtain a final co-complex score matrix. Finally, a novel graph regularized doubly stochastic matrix decomposition model is proposed to detect overlapping protein complexes from the final score matrix.

Experiment comparisons show that TINCD significantly outperforms 21 existing state-of-the-art protein complex identification methods in terms of *Accuracy* and FRAC. In addition, our model is a flexible and generic framework, which allows us to include more data sources (i.e., functional information) by simple matrix operations. When evaluating the predicted clusters over a reference set, other commonly used evaluation metrics include Sensitivity, Specificity and *f*-measure. The detailed definition of these three measures and the evaluation results of various algorithms in terms of these three measures are listed in Additional file 1. As shown in Table 1, our TINCD predicts 1,562 complexes covering 5,846 proteins, which is very close to the size of input data with 5,929 proteins. We note that the data set used in our study contains 5,929 proteins, while the three gold standard sets (i.e., CYC2008, MIPS and SGD) cover 1,324, 1,171 and 1,154 proteins. That is, the gold standard sets are far from complete. Thus, most of our predicted complexes are not able to match the benchmark complexes and TINCD achieves a low Specificity (the results are shown in Additional file 1: Table S3). However, predicted protein complexes that do not match with reference complexes are not necessarily undesired results and they would probably be novel protein complexes [15, 30]. Therefore, optimizing Specificity and *f*-measure will somehow prevent us from detecting novel complexes. On the other hand, as discussed in [15, 30], *Accuracy* and FRAC are more suitable to evaluate the performance of an overlapping protein complex detection algorithm. Furthermore, we also analyze the importance of an individual base clustering solution for TINCD, and investigate the correlation between the number of base clustering solutions and the performance of TINCD. However, since TINCD not only perform result-level integration, but also perform data-level integration, as shown in Additional file 1: Table S4, the effect of removing a base solution in result-level integration will be attenuated by the further operations in data-level integration. As shown in Additional file 1: Table S5, the performance of TINCD does not change a lot when the consensus matrix of PPI data are constructed using 5 base clustering solutions. Thus, the correlation between the number of methods and the performance of TINCD depends on the quality of the used methods. Ideally, we would think that we are able to construct more accurate consensus matrices and TINCD can generate more accurate prediction results, provided that more base clustering solutions with good performance are available. In summary, compared with existing methods, our model has the advantages as follows.

- Our TINCD model, leveraging the information from both the clustering results and raw data sources, generates more comprehensive and reliable results.
- The similarity network fusion (SNF) model, integrating heterogeneous matrices into a final co-complex score matrix, is free of scale and robust to the noise in the data.
- The graph regularized doubly stochastic matrix decomposition model considers the sparse similarities and thus ensures relatively balanced clusters.
- TINCD generates the overlapping protein complexes, which clearly reflect the biological reality on proteins’ multi-functional roles.
- Finally, TINCD is unsupervised and is thus generic enough for the integration of different types of data sources.

The computational complexity for updating *θ* in Algorithm 1 is *O*(*EK*+*NK*), where *E* is the number of non-zero items in *W*, *N* is the number of proteins in the data and *K* is the pre-defined number of complexes. Therefore the overall time cost of the graph regularized doubly stochastic matrix decomposition model is *O*((*E*+*N*)*KIter*), where *Iter* is the number of iterations. In the experiments, we implement the algorithm using Matlab in a laptop with Intel 2 CPU (2.10 GHz × 2) and 12 GB RAM. The time cost of calculating the final co-complex score matrix is at most 785 seconds (since the efficiency of SNF has been discussed in [42], we do not discuss its computational complexity here). Each update of *θ* costs at most 21 seconds and the entire estimation takes less than 4,200 seconds when the maximum number of iterations is set to 200. Frankly, our TINCD has a relatively higher computational cost compared with some base solutions. However, we would think that the running time for TINCD is still affordable for the following reasons. First, our primary task is to predict protein complexes with better accuracy and coverage. To achieve this goal, we integrate multiple data sources for clustering and it makes sense that we will higher computational cost as a sacrifice. Second, as discussed in [40], in the context of understanding and exploiting the structure of PPI networks, cluster analysis is usually used as an “offline” process to provide a comprehensive set of clustering results. It is thus acceptable that “offline”, processes have longer running time. Third, PPI data is indeed growing in recent years. The computing power of hardware (e.g., multiple CPU cores) is also under a rapid development. Moreover, we can also consider to parallelize the integration process for speedup.

Regarding the future works, we plan to design an algorithm that could incorporate other data sources (i.e., functional or structural information of proteins) [34] in addition to protein interaction data for protein complex detection. We would expect higher prediction accuracy by considering more features for proteins.

## Availability of supporting data

The datasets supporting the results of this article are included within its additional files. All the experimental results and code can be downloaded from https://github.com/Oyl-CityU/TINCD.

## Additional file

Additional file 1
**Supplementary figures and text.** This section provides the supplementary figures referred in the main text and some text which describes the detailed inference of the solution to TINCD and several biological case studies.
