# Supplementary material for: A two-layer integration framework for protein complex detection
Source: BMC Bioinformatics. 2016 Feb 24;17:100. doi: 10.1186/s12859-016-0939-3 (PMC4765032; doi:10.1186/s12859-016-0939-3)
Supplement: Additional file 1 — Supplementary figures and text. This section provides the supplementary figures referred in the main text and some text which describes the detailed inference of the solution to TINCD and several biological case studies. [file 12859_2016_939_MOESM1_ESM.pdf]

A Two-Layer Integration Framework for Protein Complex Detection

Le Ou-Yang, Wu Min, Xiao-Fei Zhang, Dao-Qing Dai, Xiao-Li Li and Hong Yan

1 Supplementary Table

Table S1: Comparison between TINCD and base clustering solutions.

			CYC2008		MIPS		SGD	
Methods	# complexes	# proteins	Acc	FRAC	Acc	FRAC	Acc	FRAC
TINCD	1562	5846	0.776	0.813	0.611	0.685	0.740	0.742
EC-BNMF	457	2105	0.751	0.677	0.617	0.671	0.716	0.623
CMBI	618	1041	0.459	0.349	0.442	0.404	0.450	0.347
InteHC	684	3400	0.748	0.634	0.645	0.670	0.712	0.597
CFinder	245	2008	0.518	0.319	0.514	0.330	0.521	0.288
CMC	562	1651	0.643	0.655	0.528	0.660	0.622	0.614
COACH	746	1838	0.650	0.664	0.559	0.665	0.631	0.631
ClusterONE	342	1366	0.584	0.438	0.493	0.448	0.583	0.445
DPClus	651	2140	0.639	0.680	0.529	0.660	0.624	0.619
IPCA	816	1621	0.617	0.575	0.525	0.601	0.610	0.547
MCL	600	4101	0.644	0.536	0.523	0.468	0.617	0.470
MCODE	108	666	0.485	0.311	0.425	0.315	0.495	0.297
RNSC	541	2095	0.619	0.506	0.501	0.458	0.619	0.508
RRW	248	1174	0.571	0.511	0.478	0.512	0.557	0.436
SPICi	412	2113	0.607	0.502	0.515	0.483	0.596	0.483
BT	409	1286	0.728	0.591	0.600	0.552	0.700	0.593
C2S	1035	4500	0.761	0.664	0.599	0.586	0.716	0.610
CACHET	449	964	0.674	0.553	0.563	0.542	0.656	0.517
Hart	390	1307	0.720	0.600	0.587	0.576	0.692	0.572
Pu	400	1504	0.732	0.579	0.604	0.567	0.695	0.581

Table S2: The Mapped Complexes for DNA-directed RNA polymerase II, DASH, RSC and Prefoldin Complexes Predicted by Various Methods

Methods	DNA-directed RNA polymerase II complex (12 proteins)		DASH complex (10 proteins)		RSC complex (17 proteins)		Prefoldin complex (6 proteins)	
	Predicted size	Overlap	Predicted size	Overlap	Predicted size	Overlap	Predicted size	Overlap
EC-BNMF	10	8	8	8	13	13	6	5
InteHC	9	7	7	7	10	10	5	5
TINCD	12	10	11	10	14	14	7	6

2 Supplementary Figure

Table S3: Comparison between TINCD and base clustering solutions in terms of Specificity, Sensitivity and f-measure.

Methods	CYC2008			MIPS			SGD		
	Specificity	Sensitivity	f-measure	Specificity	Sensitivity	f-measure	Specificity	Sensitivity	f-measure
TINCD	0.211	0.855	0.338	0.144	0.759	0.242	0.176	0.788	0.288
EC-BNMF	0.335	0.668	0.446	0.228	0.627	0.334	0.284	0.594	0.385
CMBI	0.395	0.615	0.481	0.298	0.603	0.399	0.340	0.577	0.428
InteHC	0.339	0.590	0.431	0.246	0.573	0.344	0.303	0.539	0.388
CFinder	0.294	0.310	0.302	0.184	0.249	0.211	0.261	0.276	0.268
CMC	0.267	0.649	0.378	0.187	0.603	0.285	0.235	0.592	0.336
COACH	0.345	0.765	0.475	0.248	0.737	0.371	0.296	0.718	0.419
ClusterONE	0.336	0.438	0.381	0.205	0.448	0.281	0.304	0.445	0.361
DPClus	0.273	0.704	0.394	0.187	0.639	0.290	0.223	0.617	0.327
IPCA	0.319	0.722	0.442	0.221	0.690	0.334	0.278	0.680	0.395
MCL	0.198	0.522	0.287	0.125	0.410	0.192	0.163	0.439	0.238
MCODE	0.574	0.277	0.373	0.417	0.245	0.308	0.500	0.245	0.329
RNSC	0.198	0.480	0.280	0.122	0.375	0.184	0.176	0.450	0.253
RRW	0.411	0.470	0.439	0.274	0.407	0.328	0.339	0.387	0.361
SPICi	0.248	0.466	0.323	0.163	0.390	0.229	0.216	0.422	0.286
BT	0.579	0.541	0.559	0.364	0.438	0.398	0.554	0.529	0.541
C2S	0.176	0.622	0.275	0.102	0.472	0.167	0.156	0.556	0.243
CACHET	0.745	0.719	0.732	0.526	0.671	0.590	0.615	0.661	0.637
Hart	0.578	0.550	0.564	0.362	0.456	0.403	0.513	0.502	0.507
Pu	0.519	0.519	0.519	0.364	0.460	0.407	0.505	0.512	0.509

Table S4: The performance of TINCD when one of the base clustering results is not used to construct the consensus matrix.

Methods	CYC2008		MIPS		SGD	
	Acc	FRAC	Acc	FRAC	Acc	FRAC
CFinder	0.769	0.770	0.612	0.700	0.733	0.703
CMC	0.769	0.749	0.609	0.675	0.731	0.682
COACH	0.771	0.796	0.605	0.685	0.733	0.728
ClusterONE	0.768	0.753	0.608	0.655	0.733	0.699
DPClus	0.774	0.762	0.610	0.675	0.735	0.695
IPCA	0.776	0.766	0.608	0.675	0.739	0.716
MCL	0.767	0.745	0.603	0.645	0.725	0.669
MCODE	0.766	0.783	0.604	0.660	0.730	0.725
RNSC	0.773	0.762	0.608	0.655	0.735	0.708
RRW	0.775	0.774	0.609	0.655	0.731	0.699
SPICi	0.773	0.787	0.614	0.685	0.736	0.725
BT	0.774	0.766	0.610	0.645	0.736	0.708
C2S	0.789	0.830	0.626	0.695	0.736	0.742
CACHET	0.772	0.779	0.607	0.645	0.735	0.700
Hart	0.771	0.770	0.609	0.645	0.736	0.716
Pu	0.770	0.787	0.607	0.660	0.729	0.691

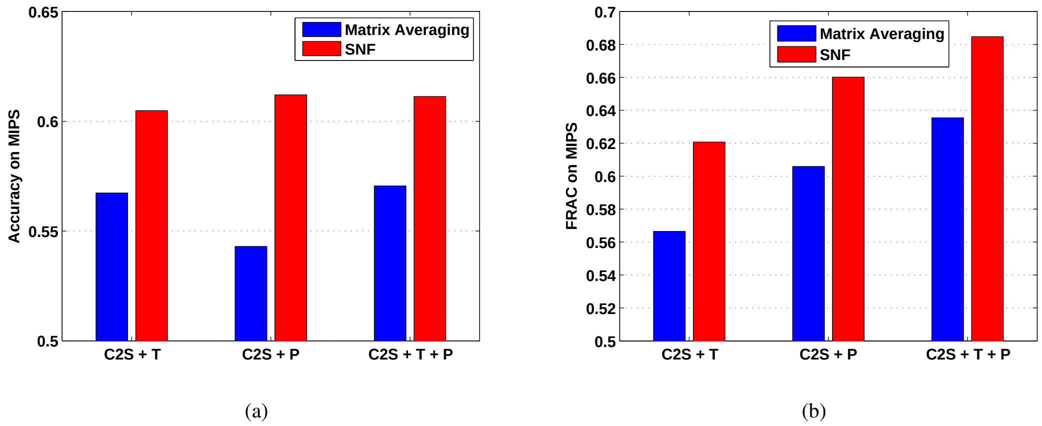

Figure S1: SNF vs. Matrix Averaging in terms of (a) Accuracy and (b) FRAC with respect to MIPS.

3 Supplementary Text

3.1 Graph regularized Doubly Stochastic Matrix Decomposition model

The objective function of Graph regularized Doubly Stochastic Matrix Decomposition model is as follows:

Table S5: The performance of TINCD when the consensus matrix of PPI data is constructed by randomly selecting 5 from the 11 base clustering solutions.

	CYC2008		MIPS		SGD	
Case	Acc	FRAC	Acc	FRAC	Acc	FRAC
1	0.774	0.757	0.611	0.675	0.735	0.720
2	0.772	0.787	0.607	0.665	0.730	0.729
3	0.784	0.834	0.617	0.700	0.737	0.742
4	0.770	0.770	0.617	0.670	0.735	0.703
5	0.763	0.774	0.602	0.645	0.729	0.720
6	0.761	0.745	0.607	0.620	0.727	0.690
7	0.768	0.774	0.604	0.635	0.726	0.711
8	0.770	0.757	0.602	0.645	0.721	0.665
9	0.772	0.745	0.607	0.694	0.731	0.703
10	0.775	0.782	0.613	0.674	0.731	0.708

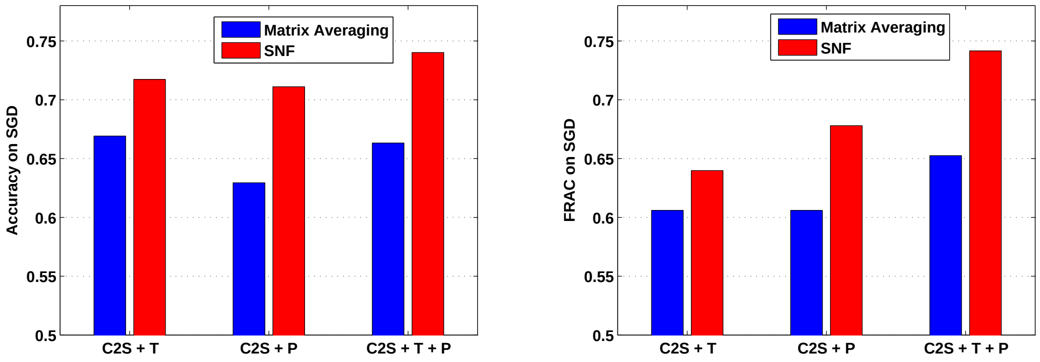

(a) (b)

Figure S2: SNF vs. Matrix Averaging in terms of (a) Accuracy and (b) FRAC with respect to SGD.

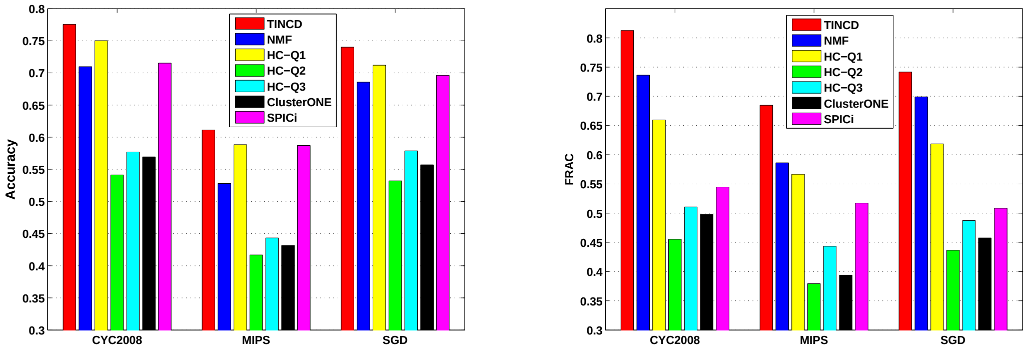

(a) (b)

Figure S3: Accuracy and FRAC of TINCD, NMF, Hierarchical clustering with 3 different quality functions (i.e., HC-Q1, HC-Q2 and HC-Q3), ClusterONE and SPICi.

$$\begin{cases} \min\limits_{\theta \geq 0} \mathcal{J}(\theta) = \sum_{ij} \left(-W_{ij} \log \hat{W_{ij}} + \hat{W}_{ij} \right) + \lambda \left(Tr(\theta^T D \theta) - Tr(\theta^T W \theta) \right). \\ s.t. \ \sum_{k=1}^{K} \theta_{ik} = 1, i = 1, \ldots, N. \end{cases} \quad (1)$$

where $\lambda \geq 0$ is the tradeoff parameters that control the balance between the two factors.

To implement the optimization, we employ a relaxed Majorization-Minimization algorithm [17]. Let $\Phi = [\phi_{iz}]$ be the Lagrange multipliers for constraint $\theta \geq 0$ and η_i be the Lagrange multipliers for constraint $\sum_{k=1}^{K} \theta_{ik} = 1$. Therefore, the

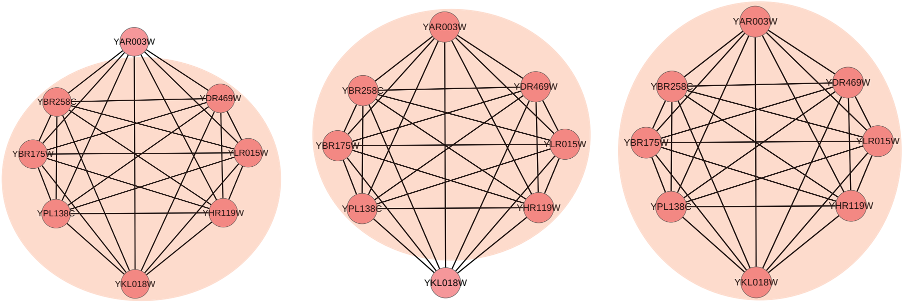

(a) EC-BNMF (b) InteHC (c) TINCD

Figure S4: The COMPASS complex as detected by different computational methods. The shadow area shows the complex predicted by each method, red circle nodes represent subunits of the COMPASS complex in CYC2008.

Lagrange function $\mathcal{L}$ is as follows:

$$\begin{aligned} \mathcal{L}(\theta, \Phi, \eta) &= \sum_{ij} \left(-W_{ij} \log \hat{W}_{ij} + \hat{W}_{ij} \right) + \lambda \left(Tr(\theta^T D \theta) - Tr(\theta^T W \theta) \right) \\ &+ \sum_{i=1}^{N} \sum_{k=1}^{K} \phi_{ik} \theta_{ik} + \sum_{i=1}^{N} \eta_i \left(\sum_{k=1}^{K} \theta_{ik} - 1 \right). \end{aligned} \tag{2}$$

Let $\nabla = \nabla^{+} - \nabla^{-}$ denote the gradient of $\mathcal{J}$ with respect to θ, where ∇^{+} and ∇^{-} are the positive and negative parts respectively. This suggests a fixed-point update rule for θ:

$$\theta' = \theta \frac{\nabla_{ik}^{-} - \eta_i}{\nabla_{ik}^{+}}. \tag{3}$$

Imposing $\sum_{k=1}^{K} \theta_{ik} = 1$, we could obtain:

$$\eta_i = \frac{b_i - 1}{a_i}. \tag{4}$$

where $a_i = \sum_v \frac{\theta_{iv}}{\nabla_{iv}^{+}}$ and $b_i = \sum_v \theta_{iv} \frac{\nabla_{iv}^{-}}{\nabla_{iv}^{+}}$.

The update rule for θ is shown in Algorithm 1. Once θ is initialized, we update θ according to Algorithm 1 until a stopping criterion is satisfied. In this study, we stop the iteration until the relative change of objective function is less than 1e-6 or the number of iterations reach the maximum iteration times (here we limit the maximum iteration times to be 200). Since the objective function in Equation (1) is non-convex, the final estimators of each θ depends on the initial values. To reduce the risk of local minimization, we repeat the entire updating procedure 20 times with random initialization and choose the result that gives the lowest value of the objective function (1) as the final estimator of θ, which is denoted as $\hat{\theta}$.

3.2 Competing methods

In the experiments, the consensus matrices are built via integrating various base clustering results from PPI data and TAP data. In particular, 11 state-of-the-art approaches were applied to PPI data to generate complexes, including CFinder [1], CMC [10], COACH [15], ClusterONE [12], DPClus [2], IPCA [9], MCL [4], MCODE [3], RNSC [8], RRW [11] and SPICi [7]. We also collected the complexes predicted from TAP data by 5 existing methods, including BT [5], C2S [16], CACHET [14], Hart [6] and Pu [13]. Protein complexes predicted by these 5 methods are obtained via the best tuned parameters and downloaded from `http://www.ntu.edu.sg/home/zhengjie/data/InteHC/`.

Among these algorithms, the performance of CFinder is determined by the size of k-clique. CMC has two key parameters called overlap threshold and merging threshold. COACH has one key parameters called ω. DPClus uses two parameters D_{in} and CP_{in} (D_{in} is a value of minimum density and CP_{in} is a minimum value for cluster property) to determine whether a neighbor should be added to the cluster. IPCA has two key parameters called T_{in} and d. MCL has one tuning parameter called inflation. MCODE has one key parameter called node score cutoff. The performance of RRW is determined by the minimum cluster size. SPICi has one key parameter called density threshold. EC-BNMF is an ensemble clustering algorithm which has two key parameters. In this study, optimal parameters are set for CFinder, CMC, COACH, DPClus, IPCA, MCL, MCODE, RRW, SPICi and EC-BNMF to generate their best results while ClusterONE and RNSC have used the default parameters set by the authors. For CFinder, k is taking a value from 3 to 10, step size by 1, and it gets the best performance when $k = 3$. For CMC, the value of the overlap threshold is from 0.2 to 0.8, with a step size of 0.1, while the value of the merging threshold is from 0 to 1, with a step size of 0.1, and it achieves the best performance when both overlap threshold and merging threshold are set to 0.5. For COACH, the values of ω is set to 0.225. For DPClus, we try different values of D_{in} and CP_{in} (from 0.3 to 0.8 with 0.1 as the step size), and it gets the best performance when both D_{in} is set to 0.6 and CP_{in} is set to 0.5. For IPCA, the value of d is set to 2, while the value of T_{in} is ranged from 0.1 to 0.9 with 0.1 as the step

Algorithm 1   Pseudocode for detecting protein complexes using graph regularized doubly stochastic matrix decomposition model.

- **Input:**
  co-complex similarity matrix W, parameters K, λ.
- **Output:**
  Q. // The set of predicted protein complexes.

begin:

t=1;

Initialize matrix θ randomly; // Initialization

while $|\frac{\mathcal{J}^{(t-1)}-\mathcal{J}^{(t)}}{\mathcal{J}^{(t)}}| > \varepsilon$ and $t \leq MaxIterations$ **do**

$s_k = \sum_{z=1}^{N} \theta_{zk}$

$Z_{ij} = \left(\sum_{k=1}^{K} \frac{\theta_{ik}\theta_{jk}}{s_k}\right)^{-1} W_{ij}$

$\nabla_{ik}^{+} = (\theta^T Z\theta)_{kk} s_k^{-2} + 2\sum_{j=1}^{N} \theta_{jk} s_k^{-1} + 2\lambda(D\theta)_{ik}$

$\nabla_{ik}^{-} = 2(Z\theta)_{ik} s_k^{-1} + \sum_{i,j=1}^{N} \theta_{ik}\theta_{jk} s_k^{-2} + 2\lambda(W\theta)_{ik}$

$a_i = \sum_v \frac{\theta_{iv}}{\nabla_{iv}^{+}}$, $b_i = \sum_v \theta_{iv} \frac{\nabla_{iv}^{-}}{\nabla_{iv}^{+}}$

$\theta_{ik} \leftarrow \theta_{ik} \frac{\nabla_{ik}^{-} a_i + 1}{\nabla_{ik}^{+} a_i + b_i}$

t = t + 1;

$\mathcal{J}^{(t)} = \sum_{ij} \left(W_{ij} \log \frac{W_{ij}}{\hat{W}_{ij}} - W_{ij} + \hat{W}_{ij}\right) + \lambda \left(Tr(\theta^T D\theta) - Tr(\theta^T W\theta)\right)$;

end while

Obtain the final protein-complex assignment matrix $\theta^{\star}$.

Output: Q, the set of predicted protein complexes.

size, and it achieves the best performance when $T_{in} = 0.5$. For MCL, the value of inflation is chosen from 1.2 to 4.9 with an interval of 0.1, and it gets the best performance when inflation is set to 1.9. For MCODE, the value of node score cutoff is searched from 0.1 to 1 with an interval of 0.1, and it gets the best performance when the node score cutoff is set to 0.2. For RRW, the minimum cluster size is set to 5. For SPICi, we try different values of density threshold, ranges from 0.1 to 1 with 0.1 increment, and it achieves the best performance when the density threshold is set to 0.5. As an ensemble clustering algorithm, the input data for EC-BNMF are a series of base clustering results which could be derived from different clustering algorithms. Therefore, in this study, we use the clustering results of the above 16 approaches as the input data for EC-BNMF. The clustering results of EC-BNMF are obtained over the best tuned parameters ($K = 2000$, $a = 2$, $b = 180$). The source codes for all these algorithms are obtained from the web pages provided in the corresponding papers.

3.3 Comparative results with respect to f-measure

When evaluating the predicted clusters set over a reference set, other commonly used evaluation metrics include Sensitivity, Specificity and f-measure. Given x_i and y_j, we consider them to be matching if $\frac{|x_i \cap y_j|^2}{|x_i||y_j|} \geq \omega$ and ω is set as 0.2 in our study. Let TP (true positive) be the number of the predicted complexes matched by the known complexes, and FN (false negative) be the number of the known complexes that are not matched by the predicted complexes, and FP (false positive) be the number of predicted complexes minus TP. Sensitivity, Specificity and f-measure are then defined as follows:

$$Sensitivity = \frac{TP}{TP + FN}, Specificity = \frac{TP}{TP + FP},$$
$$f - measure = \frac{2 \times Sensitivity \times Specificity}{Sensitivity + Specificity}. \quad (5)$$

We calculate the Sensitivity, Specificity and f-measure for each method in Table S3. As shown in Table S1, our TINCD predicts 1,562 complexes covering 5,846 proteins, which is very close to the size of input data with 5,929 proteins. However, with respect to all the three gold standard sets, our method gets low Specificity and high Sensitivity. We note that the data set used in our study contains 5,929 proteins, while the three gold standard sets (i.e., CYC2008, MIPS and SGD) cover 1,324, 1,171 and 1,154 proteins. That is, the gold standard sets are far from complete. Thus, most of our predicted complexes are not able to match the benchmark complexes. According to the definition of Specificity, these predicted complexes are treated as false positive, so TINCD achieves a low Specificity. However, predicted protein complexes that do not match with reference complexes are not necessarily undesired results and they would probably be novel protein complexes [16, 12]. Therefore, optimizing Specificity and f-measure will somehow prevent us from detecting novel complexes. This is the main reason why we do not use these metrics to evaluate the performance of various methods. On the other hand, as discussed in [16, 12], *Accuracy* and FRAC are more suitable to evaluate the performance of an overlapping protein complex detection algorithm. So we use these two metrics to evaluate the performance of various methods.

3.4 Protein complexes more accurately detected by TINCD

In this section, we will introduce several example protein complexes that are more accurately detected by TINCD. As mentioned in the manuscript, EC-BNMF and InteHC are two integrative methods that can always achieve superior performance than other computational methods, so we only list the results of TINCD, EC-BNMF and InteHC. Figure S4 shows how the COMPASS complex is found by the clustering algorithms we have studied. This complex in CYC2008 involves 8 proteins. TINCD is the only algorithm that could correctly cover all the proteins in this complex. All other algorithms make various mistakes as follows. EC-BNMF and InteHC are designed to integrate either different clustering results or diverse data sources for protein complex detection. Both of them missed 1 proteins in the COMPASS complex. In Table S2, we list more example protein complexes that are more accurately detected by TINCD.

References

[1] B. Adamcsek, G. Palla, I.J. Farkas, I. Derényi, and T. Vicsek. Cfinder: locating cliques and overlapping modules in biological networks. *Bioinformatics*, 22(8):1021 – 1023, 2006.

[2] M. Altaf-Ul-Amin, Y. Shinbo, K. Mihara, K. Kurokawa, and S. Kanaya. Development and implementation of an algorithm for detection of protein complexes in large interaction networks. *BMC Bioinformatics*, 7(1):207, 2006.

[3] Gary D Bader and Christopher WV Hogue. An automated method for finding molecular complexes in large protein interaction networks. *BMC Bioinformatics*, 4(1):2, 2003.

[4] Anton J Enright, Stijn Van Dongen, and Christos A Ouzounis. An efficient algorithm for large-scale detection of protein families. *Nucleic Acids Res.*, 30(7):1575–1584, 2002.

[5] Caroline C Friedel, Jan Krumsiek, and Ralf Zimmer. Bootstrapping the interactome: unsupervised identification of protein complexes in yeast. *Journal of Computational Biology*, 16(8):971–987, 2009.

[6] G Traver Hart, Insuk Lee, and Edward M Marcotte. A high-accuracy consensus map of yeast protein complexes reveals modular nature of gene essentiality. *BMC Bioinformatics*, 8(1):236, 2007.

[7] Peng Jiang and Mona Singh. Spici: a fast clustering algorithm for large biological networks. *Bioinformatics*, 26(8):1105 – 1111, 2010.

[8] AD King, N. Pržulj, and I. Jurisica. Protein complex prediction via cost-based clustering. *Bioinformatics*, 20(17):3013–3020, 2004.

[9] Min Li, Jian-er Chen, Jian-xin Wang, Bin Hu, and Gang Chen. Modifying the dpclus algorithm for identifying protein complexes based on new topological structures. *BMC Bioinformatics*, 9(1):398, 2008.

[10] Guimei Liu, Limsoon Wong, and Hon Nian Chua. Complex discovery from weighted ppi networks. *Bioinformatics*, 25(15):1891–1897, 2009.

[11] Kathy Macropol, Tolga Can, and Ambuj K Singh. Rrw: repeated random walks on genome-scale protein networks for local cluster discovery. *BMC Bioinformatics*, 10(1):283, 2009.

[12] Tamás Nepusz, Haiyuan Yu, and Alberto Paccanaro. Detecting overlapping protein complexes in protein-protein interaction networks. *Nature Methods*, 9(5):471–472, 2012.

[13] Shuye Pu, Jim Vlasblom, Andrew Emili, Jack Greenblatt, and Shoshana J Wodak. Identifying functional modules in the physical interactome of saccharomyces cerevisiae. *Proteomics*, 7(6):944–960, 2007.

[14] Min Wu, Xiao-li Li, Chee-Keong Kwoh, See-Kiong Ng, and Limsoon Wong. Discovery of protein complexes with core-attachment structures from tandem affinity purification (tap) data. *Journal of Computational Biology*, 19(9):1027–1042, 2012.

[15] Min Wu, Xiaoli Li, Chee-Keong Kwoh, and See-Kiong Ng. A core-attachment based method to detect protein complexes in ppi networks. *BMC Bioinformatics*, 10(1):169, 2009.

[16] Zhipeng Xie, Chee Keong Kwoh, Xiao-Li Li, and Min Wu. Construction of co-complex score matrix for protein complex prediction from ap-ms data. *Bioinformatics*, 27(13):i159–i166, 2011.

[17] Zhirong Yang and Erkki Oja. Clustering by low-rank doubly stochastic matrix decomposition. In *Proceedings of the 29th International Conference on Machine Learning (ICML-12)*, pages 831–838, 2012.
